# Stroke-like episodes in coenzyme-Q deficiency may respond to NO-precursors and non-mitochondrion-toxic antiepileptic drugs

**DOI:** 10.1016/j.ymgmr.2018.10.003

**Published:** 2018-10-18

**Authors:** Josef Finsterer

**Affiliations:** Krankenanstalt Rudolfstiftung, Messerli Institute, Veterinary University of Vienna, Vienna, Austria

**Keywords:** Coenzyme-Q, Respiratory chain, Epilepsy, Seizures, Antioxidants, Mitochondrion-toxic

Letter to the Editor

With interest we read the article by Bosch et al. about a family with coenzyme-Q deficiency due to a mutation in the *COQ4* gene [1]. We have the following comments.

Patient-2 obviously experienced a stroke-like episode (SLE) at age 13 y, clinically manifesting as headache, vomiting, impaired consciousness, and hemianopsia, as documented on MRI [1]. Since there are indications that NO-precursors are beneficial for SLEs [2], it would be interesting to know if the reported patient received l-arginine or l-citrulline intravenously at onset of the SLE. We should also be informed if impaired consciousness was due to non-convulsive seizure activity on EEG, since the patient had developed tonic-clonic seizures since age 9 y [1]. Even seizures during SLEs have been reported to respond favourably to l-arginine [3].

Astonishingly, the patient received carbamazepine (CBZ) for seizures since age 9 y [1]. From CBZ it is well known that it can be mitochondrion toxic [4]. Thus, it is conceivable that tremor and the “spinocerebellar syndrome”, developing 1 y after starting CBZ, could be a side effect of CBZ. It is even possible that the SLE at age 13 y was triggered by the toxic effect of CBZ. Thus, we should know the dosage of CBZ and if serum levels of CBZ were outside the therapeutic range. Cerebral imaging did not show cerebellar atrophy and thus does not explain these clinical abnormalities.

In patient-1 a tectal glioma was suspected at age 5 y, being treated with radiotherapy [1]. We should be informed if the suspicion of a glioma was confirmed on MR-spectroscopy or cerebral PET investigations. Since coenzyme-Q deficiency frequently is associated with lactic acidosis in blood and CSF [5], we should know if occurrence of seizures or SLEs correlated with these lactate levels.

In summary, the reported patients may profit from adding NO-precursors, from replacement of CBZ by a non-mitochondrion toxic antiepileptic drug, and from measuring serum/CSF lactate.

## Conflict of interest

There are no conflicts of interest.

## Funding

No funding was received.
